# csdR, an R package for differential co-expression analysis

**DOI:** 10.1186/s12859-022-04605-1

**Published:** 2022-02-19

**Authors:** Jakob P. Pettersen, Eivind Almaas

**Affiliations:** 1 Department of Biotechnology and Food Science, NTNU- Norwegian University of Science and Technology, Trondheim, Norway; 2 K.G. Jebsen Center for Genetic Epidemiology, Department of Public Health and General Practice, NTNU- Norwegian University of Science and Technology, Trondheim, Norway

**Keywords:** R, Genome-scale, Co-expression, Gene network, Network

## Abstract

**Background:**

Differential co-expression network analysis has become an important tool to gain understanding of biological phenotypes and diseases. The CSD algorithm is a method to generate differential co-expression networks by comparing gene co-expressions from two different conditions. Each of the gene pairs is assigned conserved (C), specific (S) and differentiated (D) scores based on the co-expression of the gene pair between the two conditions. The result of the procedure is a network where the nodes are genes and the links are the gene pairs with the highest C-, S-, and D-scores. However, the existing CSD-implementations suffer from poor computational performance, difficult user procedures and lack of documentation.

**Results:**

We created the R-package csdR aimed at reaching good performance together with ease of use, sufficient documentation, and with the ability to play well with other tools for data analysis. csdR was benchmarked on a realistic dataset with 20,645 genes. After verifying that the chosen number of iterations gave sufficient robustness, we tested the performance against the two existing CSD implementations. csdR was superior in performance to one of the implementations, whereas the other did not run. Our implementation can utilize multiple processing cores. However, we were unable to achieve more than $\sim$2.7 parallel speedup with saturation reached at about 10 cores.

**Conclusion:**

The results suggest that csdR is a useful tool for differential co-expression analysis and is able to generate robust results within a workday on datasets of realistic sizes when run on a workstation or compute server.

## Introduction

Experimental high-throughput techniques, such as microarray and RNA sequencing, allow for large-scale assays of gene expressions. Correlation-based network approaches have been used for analysing a wide variety of gene-expression data in humans, identifying both individual genes and clusters of genes with prominent relationships to the phenotype (disease) in question [1–4]. More recently, there has been a realization that differential co-expression analyses, i.e. the study of changes in the correlations rather than just a test for their presence or absence in the conditions, may identify important genes [5, 6]. This may be of interest for the study of diseases, as a central goal is to identify genes contributing to differences between sick patients and healthy controls.

There exist multiple methods for differential co-expression analysis [7, 8]. Some make separate co-expression networks for both conditions and compare the networks in order to score differential expressed genes [9–11]. Another major approach is based on scoring gene pairs directly based on their differential expression between different conditions [12–15]. The CSD approach [7] is of the second type and explicitly distinguishes between three different kinds of differential co-expression, that of Conserved (C), Specific (S), and Differentiated (D), hence its name. Each pair of genes will have a score for each of these three categories.

Previously, two implementations of CSD have been written. The first one (https://github.com/andre-voigt/CSD) was written as part of the original CSD work [7]. It is implemented in a combination of C++ and Python and is not focused on performance and user-friendliness. The other implementation (https://github.com/magnusolavhelland/CSD-Software) is written in C++ and is fine-tuned for performance [16]. However, practical experience has shown it difficult to use due to its strict and obscure requirements for input data format. CoDiNa [17] is an R package which implements a procedure similar to CSD and allows for comparing data from more than two environments. On the other hand, CoDiNa does not account for the variability in co-expression within an environment.

## Implementation

We will assume that the expression vectors of two genes A and B have Spearman correlations of $\rho _1$ and $\rho _2$ in the first and second condition respectively. Furthermore, we define $\sigma _1$ and $\sigma _2$ as the corresponding standard deviations of the aforementioned Spearman correlations, estimated by resampling. The values for C, S and D are then defined by:

1 $$\begin{aligned} C&=\frac{\left| \rho _1+\rho _2\right| }{\sqrt{\sigma _1^2+\sigma _2^2}}, \end{aligned}$$

2 $$\begin{aligned} S&=\frac{\left| \left| \rho _1\right| -\left| \rho _2\right| \right| }{\sqrt{\sigma _1^2+\sigma _2^2}}, \end{aligned}$$

3 $$\begin{aligned} D&=\frac{\left| \left| \rho _1\right| +\left| \rho _2\right| -\left| \rho _1+\rho _2\right| \right| }{\sqrt{\sigma _1^2+\sigma _2^2}}. \end{aligned}$$

The CSD algorithm consists of three principal parts:

1. Calculation of the Spearman correlation between each pair of genes for each of the two datasets individually. This is conducted with resampling to provide an estimate of the variance of the correlation.
2. Comparison of the values of mean correlation and standard deviation from the two conditions, allowing the computation of Conserved, Specific, and Differentiated scores.
3. Selection of the gene pairs with the highest values for C, S and D, and the generation of a network from them. In typical disease-network analyses, this network is studied further with tools such as module finding and enrichment analysis.

**Fig. 1:**
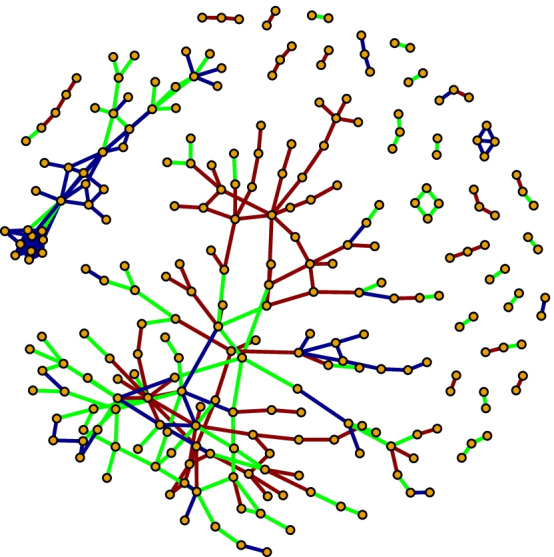
An example CSD network taken from the csdR vignette. The nodes are genes and the edges C- (dark blue), S- (green) and D-links (dark red)

An example CSD network containing both C-, S- and D-links is shown in Fig. 1. A link in a CSD-network indicates a relation between the genes across the two conditions and is likely to be due to regulatory effects which are the same or different in the two conditions. With this in mind, we can consider the CSD network a product of the underlying gene regulatory network. This allows us to suggest regulatory mechanisms which are the same for both conditions in addition to mechanisms which are different in the two conditions. Hence, CSD can be used as a tool to point to possible gene-phenotype relationships underlying the condition in question. In turn, the results from CSD can be integrated with prior knowledge to shed more light on the genetic basis for the condition and serve as a starting point for follow-up experiments.

csdR is an R [18] package which implements this procedure and is written to achieve good performance, be well documented and user-friendly, and provide seamless integration with other tools in the R ecosystem. The source code is available on GitHub (https://github.com/AlmaasLab/csdR). Parts of it are written with Rcpp [19–21] in order to boost performance. The package is designed to utilize multicore processors and processor SIMD (Single Instruction, Multiple Data) instructions through its usage of openMP [22]. In addition, the package is available in Bioconductor release 3.14.

The data provided to the package must be numerical data organized in matrices by sample and gene. In theory, any numerical measure of gene expression could be used. In practice, normalized read counts from RNA-seq or proteomics studies are the most relevant to use. Note that imputation of missing values is not implemented in the package. If missing values are present in the raw data, an error message will be reported to the user.

In the original implementation [7] and the CSD C++ implementation, the resampling is done through an ad-hoc method termed independent subsampling, meaning that no points are sampled together more than once and each subsample has a fixed size. Instead, our implementation uses bootstrapping [23] which is a more common statistical practice. This means that the data points are drawn with replacement, and each bootstrap sample contains as many points as the original sample. Consequently, a data point is likely to be picked more than once in the same sample or not being in the sample at all. Compared to indendent subsampling, bootstrapping may be conducted an arbitrary number of times, which ensures stable results given a sufficiently large number of iterations. In addition, bootstrapping is easier to implement and allows for faster computations.

As part of computing the Spearman correlation, the observational ranks of the genes in each sample must be computed. In the original implementation, this rank is re-computed for every gene pair. csdR optimizes this approach by first finding the ranks of all genes before computing the all-to-all Pearson correlation of the ranks. For this computation, the efficient WGCNA version of cor is used [1, 24]. Internally, WGCNA::cor() uses matrix multiplication handled by BLAS (Basic Linear Algebra Subprograms). Because this step is the major performance bottleneck, linking R against an optimized BLAS library, such as OpenBLAS (http://www.openblas.net/), is strongly recommended.

In order to ensure numerically stable computation of the variance of the co-expressions, Welford’s algorithm [25] is applied. For the final step of selecting edges with the largest values of C, S and D, past implementations used random sampling to find the importance cutoff. Our implementation however, uses the more direct approach of partial sorting through the C++ STL functions std::nth_element and std::sort.

## Results and discussion

For small datasets (order of 100 samples and 100 genes), all implementations are so fast that the runtime is of no practical importance. We benchmarked the different implementation on a realistic dataset derived from RNA-seq of thyroid glands. The data for the patients with thyroid cancer (case) consisted of 504 samples, while the control dataset consisted of 399 samples. These datasets are the full versions of the down-scaled datasets sick_expression and normal_expression provided in the package. See https://github.com/AlmaasLab/csdR/blob/main/inst/script/download_preprocess.md for more details on how the data were obtained and pre-processed. There were a total of 20, 645 genes being compared, which resulted in 213, 097, 690 different gene pairs. We ran the three implementations with importance level set to $p=10^{-6}$. This resulted in C-, S- and D-networks with 213 edges each. For the two first implementations, the number of random selections was kept to $10^4$, and the size of the subsamples set to 10. All benchmarked software was compiled with GCC version 9.3.0 using compiler flags -O3 -march=native and run on 10 virtual 2.4 GHz Intel Broadwell processors. For csdR, the benchmarks were conducted using R version 4.1.0 linked against libopenblas version 0.3.15.

**Fig. 2:**
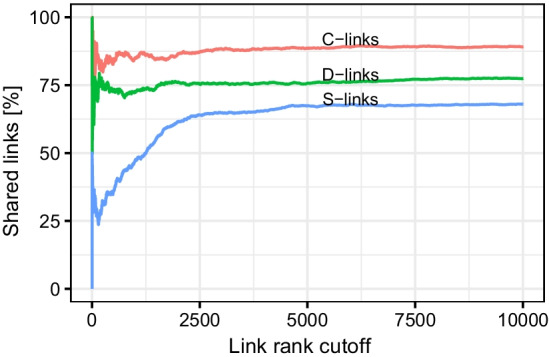
Cumulative overlap of the highest ranking links from csdR. The shared links are the intersection of 10 parallels run over 1000 iterations with different random seeds

In order to determine the number of iterations for csdR, we investigated the robustness of the highest ranking links across different random seeds. We ran 10 parallels with different random seeds over 1000 iterations, identified the intersection of the highest ranking gene pairs between these 10 parallels, and finally calculated the proportion these shared gene pairs made up (Fig. 2). For all three link types, the recall across all 10 parallels stabilised at approximately 70%, 80%, and 90% for the S-, D and C-links, respectively, when the number of selected links exceeded 2500. For smaller numbers of links, there are more random fluctuations. We observe that the S-links have the lowest rate of recall. This observation can be attributed to the fact that gene pairs with large S-values have low levels of co-expression in one of the conditions and their scores are therefore more susceptible to random noise. We are of the opinion that the robustness at 1000 iterations is sufficient for practical use, and this choice was therefore used in the benchmarking process. Better robustness may be obtained by increasing the number of iterations at the expense of run time.

For this benchmark, we were not able to use CSD-C++, as we were unable to reshape the data into a format the program would accept. A custom format repair tool (https://github.com/lars-as/csd_cs_ged_tools) was attempted, but did not resolve the issues. For the other two implementations, the results are shown in Table 1. We notice that csdR is much faster than the original implementation even on a single core. The original implementation is single-threaded and can thus not take advantage of multiple cores. For csdR, running the algorithm on 5 cores instead of one reduces the time spent approximately by a factor two. Doubling the core count to 10 provides another reduction factor of $\sim$25% of the time. For 15 cores however, no performance gain beyond the margin of error was observed. We suspect that the algorithm’s failure to scale to such a large number of cores is due to the system’s memory bandwidth being exhausted. Another result worth noticing is the fact that only the first step in the CSD procedure determines the performance in practise. The contributions from step 2 and 3 were negligible except for step 3 in the original implementation, which consumes up 8.4% of the overall time. In terms of memory usage, csdR consumed approximately 30 GB of RAM on the benchmarked datasets. Due to the fact that most laptops and many desktop computers have less memory than this, csdR is more suited for powerful workstations or compute servers.

**Table 1:** Running time (s) for CSD on the large datasets with 1000 iterations

Implementation	Cores	Step 1	Step 2	Step 3	Total
Original	1	1,261,628	4900	116,051	1,382,579
csdR	1	41,387	79	11	41,477
csdR	5	20,737	50	15	20,802
csdR	10	15,488	45	13	15,546
csdR	15	15,192	50	12	15,254

## Conclusions

We have shown that csdR is reasonably fast even for large datasets and provides sufficiently robust results. In addition, it is more accessible to the common user and better documented than the previous CSD implementations.

## Availability and requirements

- **Project name:** csdR
- **Project home page:** https://github.com/AlmaasLab/csdR
- **Operating systems:** Cross-platform
- **Programming language:** R, C++11
- **Other requirements:** R($>=4.1.0$), R packages WGCNA, glue, matrixStats, RhpcBLASctl and Rcpp
- **License:** GNU General Public License v3.0
- **Any restrictions to use by non-academics:** The terms of the GPL-3 license must be respected.

## Data Availability

The scripts and datasets used for benchmarking are available for download at Figshare 10.6084/m9.figshare.16713121

## Abbreviations

BLAS: Basic linear Algebra Subprograms
CSD: Conserved, Specific, and Differentiated
SIMD: Single instruction, multiple data
WGCNA: Weighted correlation network analysis
